# Retraction: miR-24-3p Suppresses Malignant Behavior of Lacrimal Adenoid Cystic Carcinoma by Targeting PRKCH to Regulate p53/p21 Pathway

**DOI:** 10.1371/journal.pone.0260999

**Published:** 2021-12-02

**Authors:** The PLOS ONE Editors

Following the publication of this article [1], concerns were raised regarding results presented in Figs 3, 5, 6, and S7 Fig. Specifically,

- The following panels appear similar, but represent different experimental results:
      ○ Fig 3A left ACC-2 ASO-NC and S7B Fig right ACC-2 shR-PRKCH+ASO-miR-24-3p (4) panel
      ○ Fig 3A right ACC-M pcDNA3 and S7B Fig left ACC-M shR-PRKCH+ASO-miR-24-3p (4) panel
      ○ Fig 3A right ACCM ASO-NC and S7B Fig left ACC-M shR-PRKCH+ASO-NC (3) panel
      ○ Fig 3B ACC-2 ASO-NC 0hr and Fig 3B ACC-2 ASO-miR-24-3P 0hr
      ○ Fig 3B ACC-2 pri-miR-24-3p 48hr and Fig 3B ACC-M pri-miR-24-3p 24hr
      ○ The top left GAPDH panel of Fig 5C and the bottom right GAPDH panel of Fig 5C
      ○ The right colony assay panel for pSilencer-NC+ASO-NC ofS7 Fig and the right colony assay panel for shR-PRKCH+ASO-miR-24-3p of S7 Fig.
- The following panels partially overlap, but represent different experimental results:
      ○ Fig 3A left ACC-M pcDNA3, S7B Fig right ACC-M pSilencer-NC+ASO-NC (1) panel, and S7B Fig right ACC-M shR-PRKCH+ASO-miR-24-3p (4) panel
      ○ The left ACC-2 pcDNA3+pcDNA3/PRKCH (2) panel of Fig 6D and the left ACC-M pcDNA3+pcDNA3/PRKCH (2) panel of Fig 6D.
      ○ The right ACC-2 pcDNA3+pcDNA3 (1) panel of Fig 6D and the right ACC-2 pri-miR-24-3p+pcDNA3/PRKCH (4) panel of Fig 6D.

The corresponding author explained that duplicate and partially overlapping panels were included inadvertently during figure preparation. In addition to the concerns raised by the journal, the corresponding author clarified that the Fig 5C ACC-2-GAPDH-pSilencer-NC panel, and the S7B Fig ACC-2-pSilencer-NC+ASO-NC, ACC-2-shR-PRKCH+ASO-NC, and ACC-M-pSilencer-NC+ASO-miR-24-3p panels are incorrect and were inadvertently included during the figure preparation.

The above concerns impact multiple figure panels representing results obtained from four different types of experiments reported in the article. Underlying data and replacement panels for each figure affected by the image concerns were provided to the journal. While the replacement panels satisfactorily addressed some of the above concerns, not all issues were resolved, and the *PLOS ONE* Editors remain concerned about the reliability of data reporting overall in the article given the number of errors identified. Therefore, the *PLOS ONE* Editors retract this article.

MZ, JZ, HZ, and HT agreed with the retraction. MZ, JZ, and HZ apologized for the issues with the published article.
